# Spiropyran as a potential molecular diagnostic tool for double-stranded RNA detection

**DOI:** 10.1186/s42490-019-0008-x

**Published:** 2019-03-18

**Authors:** Ahsan Ausaf Ali, Minjeong Kang, Raisa Kharbash, Yoosik Kim

**Affiliations:** Department of Chemical and Biomolecular Engineering and KI for Health Science and Technology (KIHST), Korea Advanced Institute of Science and Technology (KAIST), Daejeon, 34141 South Korea

**Keywords:** Spiropyran, Double-stranded RNA, Biosensor, UV-vis spectroscopy, DNA-demethylating agent, Drug responsiveness, Innate immune response

## Abstract

**Background:**

Long double-stranded RNAs (dsRNAs) are duplex RNAs that can induce immune response when present in mammalian cells. These RNAs are historically associated with viral replication, but recent evidence suggests that human cells naturally encode endogenous dsRNAs that can regulate antiviral machineries in cellular contexts beyond immune response.

**Results:**

In this study, we use photochromic organic compound spiropyran to profile and quantitate dsRNA expression. We show that the open form of spiropyran, merocyanine, can intercalate between RNA base pairs, which leads to protonation and alteration in the spectral property of the compound. By quantifying the spectral change, we can detect and quantify dsRNA expression level, both synthetic and cellular. We further demonstrate that spiropyrans can be used as a molecular diagnostic tool to profile endogenously expressed dsRNAs. Particularly, we show that spiropyrans can robustly detect elevated dsRNA levels when colorectal cancer cells are treated with 5-aza-2′-deoxycytidine, an FDA-approved DNA-demethylating agent used for chemotherapy, thus demonstrating the use of spiropyran for predicting responsiveness to the drug treatment.

**Conclusion:**

As dsRNAs are signature of virus and accumulation of dsRNAs is implicated in various degenerative disease, our work establishes potential application of spiropyrans as a simple spectral tool to diagnose human disease based on dsRNA expression.

**Electronic supplementary material:**

The online version of this article (10.1186/s42490-019-0008-x) contains supplementary material, which is available to authorized users.

## Background

Long double-stranded RNAs (dsRNAs) are analogous to DNAs in that they both exist in duplex helical structures. Historically, these dsRNAs are associated with virus as they are believed to be byproducts of viral replication of positive sense RNA viruses [1]. Consequently, when expressed in mammalian cells, long dsRNAs are recognized by innate immune response proteins which induce interferons, suppress translation, and initiate apoptosis programs [2]. Although dsRNAs are strongly associated with immune response to viral infection, increasing evidences suggest that human cells naturally express endogenous dsRNAs that can regulate antiviral machineries in various cellular contexts such as during the cell cycle and response to stressors [3, 4]. Recently, two research groups have independently shown that mitochondrial RNAs can exist as intermolecular dsRNAs and are recognized by immune response proteins to regulate antiviral signaling [5, 6]. Moreover, accumulation of endogenously encoded dsRNAs is related to the onset of autoimmune [7] and age-related macular degeneration [8]. dsRNAs also play a key role during cellular response to chemotherapy where treatment of the DNA-demethylating agent leads to cell death by inducing the transcription of endogenous dsRNA genes, which subsequently activate antiviral machineries [9, 10].

One interesting aspect of the dsRNA-mediated antiviral signaling is that immune response proteins recognize specific structural signatures of the RNA such as the double-stranded secondary structure rather than specific sequences of the RNA [11]. Therefore, to investigate the potential of endogenous dsRNAs as novel signaling molecules and their function during the onset of human disease, we need to develop a quantitative approach to measure the collective expression level of all types of dsRNAs with minimal sequence specificity.

Photochromic spiropyrans undergo drastic structural changes between closed spiropyran (SP) and open merocyanine (MC) isoforms. Due to their photo-switching properties, spiropyran derivatives have a wide range of applications, from design of smart nanomaterials to optical regulation of biomacromolecules [12]. Specifically, spiropyrans have been used in solution, blended in nanomaterials and polymers to develop sensors for various target molecules [13–21]. These sensors rely on the colorimetric and spectral variations in the spiropyrans occurring due to protonation by and complex formation with the target material.

Recently, numerous studies have investigated and characterized photo-switchable interactions between spiropyran derivatives and biological molecules such as DNA [22–26]. They found that only the open form can interact with DNA, which can be monitored using characteristic changes in the UV-Vis absorbance spectrum of the compound [22–26]. These studies revealed that considerable changes occur in the absorbance spectrum due to intercalation with DNA base pairs and that the degree of the change is correlated with the amount and the sequences of the DNA present.

In contrast to the spiropyran-DNA interaction, which has been under extensive investigation in recent years, little has been done to examine possible interaction between spiropyran derivatives and RNAs. Previous studies with spiropyrans have primarily focused on tertiary structured short hairpin RNAs and their qualitative interactions as aptamers via surface plasmon resonance [27] and nanopores [28]. More recently, studies utilized spectral properties of gold nanoparticles and quantum dots to detect and quantify expression of specific RNA targets such as mRNAs [29, 30], single-stranded RNAs (ssRNAs) [31, 32], and short double-stranded RNAs (dsRNAs) [33]. However, they all used RNA probes that are complementary to the target RNAs and thus, their approach is limited to detecting just one or few RNA transcripts [34].

In this study, we investigate the potential of using spiropyrans as a tool to detect and profile the overall expression of dsRNAs. Our goal is three folds: 1) To establish spiropyran as a molecule that can interact with dsRNAs, 2) To characterize the interactions between dsRNA and spiropyran, and 3) To apply spiropyran to detect changes in levels of endogenous dsRNAs in response to various stressors. We find that the open form of spiropyran, MC, can moderately interact with dsRNAs, which results in protonation of MC to MCH+ and alters the characteristic UV-Vis absorbance spectrum of the compound. By quantifying this change, we can access the amount of dsRNA present in the solution. The effect is greater for the GC pair than that of the AU pair and mixture of the two types of nucleotide pairs also show strong characteristic decrease in the light absorbance. Moreover, using RNases with different substrate specificities, we can enrich and detect long dsRNAs by removing most of the ssRNAs and short hairpin RNAs from the cell extract. Furthermore, we apply our approach to demonstrate the potential usage of MC and elevation of dsRNA expression as a predictive marker for cellular response to the DNA-demethylating agent 5-aza-2′-deoxycytidine (decitabine), which is commonly used to treat colorectal cancer and pre-leukemic disorder myelodysplasia (MDS) [9, 35]. Collectively, our work establishes spiropyran as a potential molecular diagnostic tool for dsRNA detection from human cells and serves as the first step for the development of bio-diagnostic tool based on dsRNA expression.

## Results

### Only the open MC form interacts with dsRNAs

**Fig. 1:**
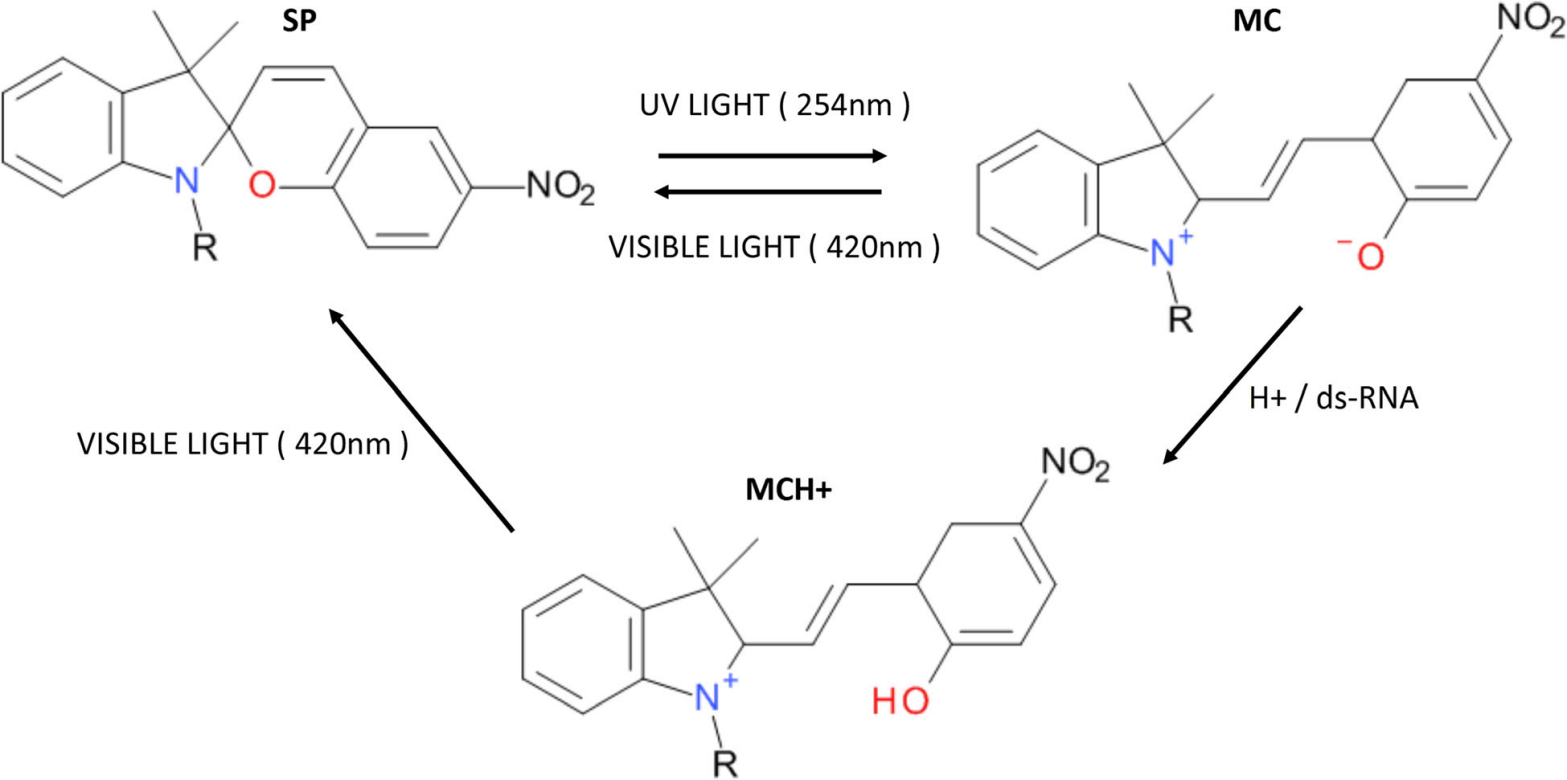
Molecular structures of the spiropyran and its isoforms. The spiropyran derivative used in this study along with its isoforms and their abbreviations. R = CH_2_CH_2_CH_2_(CH_3_)_3_N^+^

The SP and MC isoforms of spiropyrans can be converted from each other using UV and visible light, respectively (Fig. 1). They have different geometric properties, which can affect their interaction with nucleic acids, owing to the base pair stacking in the double helix structure. Previously, the MC form has been shown to undergo protonation to MCH+ while complexed with DNA at pH 7 [22–24]. In this context, the net positive charge on the spiropyran derivatives may drive the compound towards the negatively charged DNA double helix. Following intercalation, MC becomes protonated to MCH+ which is evident from the extinction coefficients of the spectra [24, 36]. We proposed that similar interactions may occur between spiropyrans and dsRNAs as dsRNAs also exist in double stranded helical structure, but at the same time, they might be less accessible because dsRNAs form the A-form helix which has narrower and shallow major grooves than DNA’s B-form helix [37].

UV-Vis spectroscopy is commonly used to study interactions between nucleic acids and potential ligands [38]. If the two species interact, it results in shift in the UV-Vis absorbance spectrum of the ligand, which can be quantified to calculate interaction parameters such as the dissociation constant. To investigate whether spiropyrans can be used to detect dsRNAs present in cell lysates, we first investigated interaction between SP and synthetic dsRNAs using UV-Vis spectroscopy. Of note, to test the physiological relevance of the synthetic dsRNAs, we transfected synthetic 100 mer poly AU to HCT116 colorectal cancer cells and confirmed that these dsRNAs can induce innate immune response (Additional file 1: Figure S2). In addition, we used triple distilled nuclear free water (TDW) to reconstitute lyophilized poly AU RNA powder. Yet, these RNAs retained their double-stranded secondary structure and when transfected into the cell, poly AU dsRNAs are recognized by immune response protein kinase RNA-activated (PKR), resulting in phosphorylation of PKR and activation of PKR signaling (Additional file 1: Figure S2). This indicates that our synthetic dsRNAs can be used to mimic natural dsRNAs and can maintain their double-stranded secondary structure in TDW. Thus, we proceeded our initial analysis using synthetic dsRNAs.

**Fig. 2:**
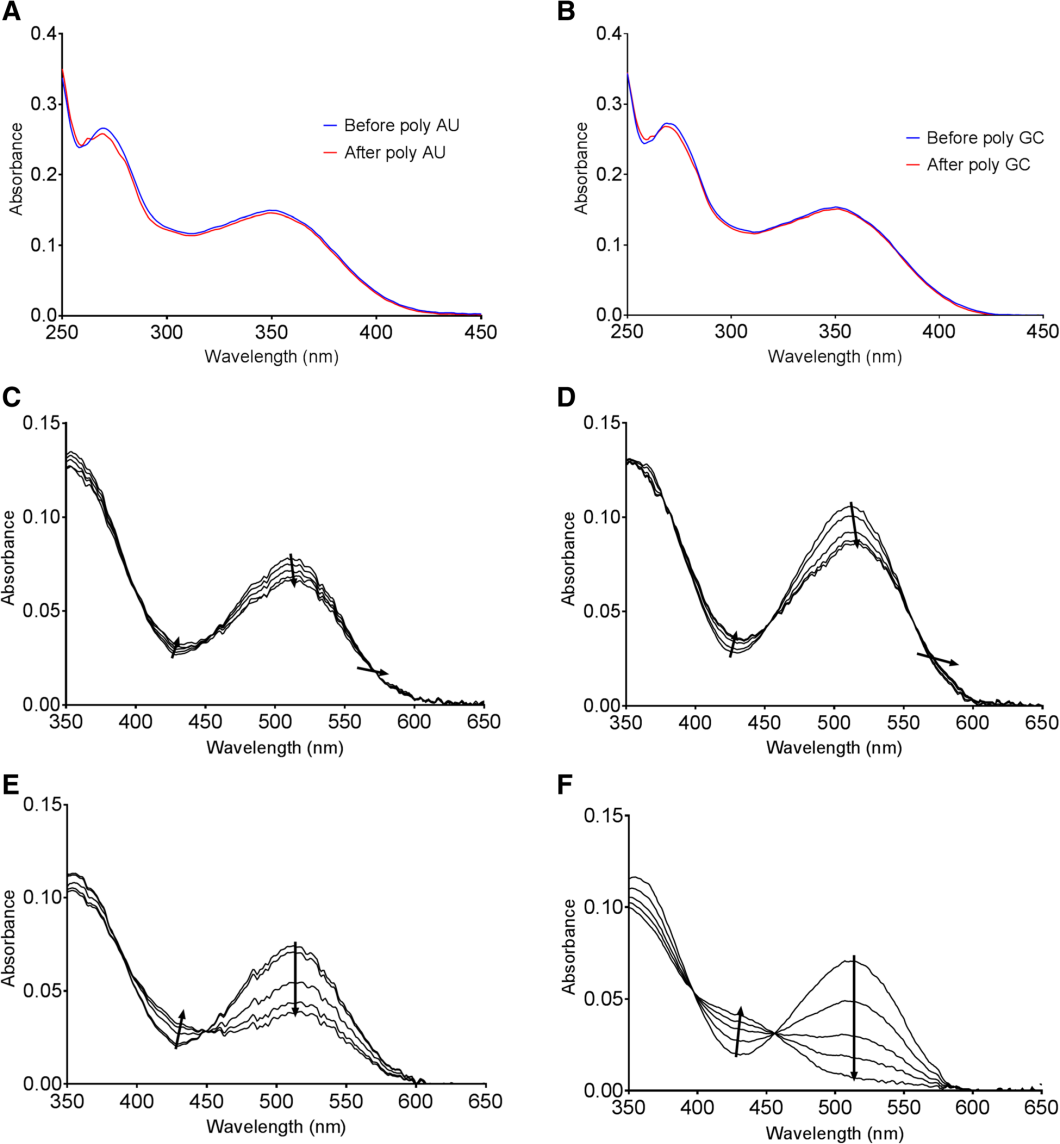
Interaction of the open MC form with dsRNAs. **a, b** The absorbance spectra before and after the addition of 200 μM of poly AU (**a**) or poly GC (**b**) to 12 μM of SP. **c, d** Change in the absorbance spectra as an increasing amount of poly AU (**c**) or poly GC (**d**) is added to 12 μM of MC in 9 mM Na + ions buffer at pH 7. Concentration is increased from 0 to ~ 600 μM. **e, f** Change in the absorbance spectra of MC upon addition of an increased amount of poly AU (**e**) or poly GC (**f**) to 12 μM of MC at pH 7 in TDW. Concentration is increased from 0 to~ 450 μM

We prepared 20 base pair long poly GC and poly AU dsRNAs and added 200 μM solution of these dsRNAs to 12 μM SP solution. In neither of the two types of dsRNAs, we observed any change in the SP’s spectrum, which could be superimposed almost perfectly with the original one without dsRNAs (Fig. 2a and b). Of note, in our analysis, we removed the absorbance of dsRNAs from the sample to analyze only the SP spectrum. SP has a non-planar structure [25] which likely prevents it from intercalating with the RNA base pairs thereby resulting in no spectral changes as also observed in the case of DNA [22].

Next, we examined the interaction between dsRNAs with the open form, MC. To induce isomerization of SP to MC, we irradiated the SP solution with UV light to produce an equilibrium solution containing about (63/37) ±5% ratio of SP to MC with a quantum yield of ring opening reaction of 0.015 and ring closing of 0.014. In the presence of MC, addition of dsRNAs resulted in noticeable change in the absorbance spectra (Fig. 2c and d). Both addition of poly AU and poly GC resulted in a significant hypochromic shift at 512 nm and a red-shift towards the leading shoulder. Both of these respective shifts in the spectra are signatures of intercalation [39–44]. Moreover, similar shifts in the MC spectra have been reported when MC is incubated with DNA [22], which further suggests that MC can intercalate between RNA base pairs. In addition, we also observed a smaller but consistent increase at 432 nm absorbance peak accompanied by two surrounding isosbestic points. This change can be accounted by the conversion of the MC to its protonated MCH+ form, which is considered to be more stable between nucleic acid base pairs [22]. Changes observed for poly AU are noticeably weaker (both the red-shift and the hypochromic shift) than those for poly GC indicating that MC has a preference for poly GC. Estimated extinction coefficients for these species including isosbestic points and hypochromic shift and red-shift are shown in Additional file 1: Figure S3, which further confirm the presence of the different binding species.

**Fig. 3:**
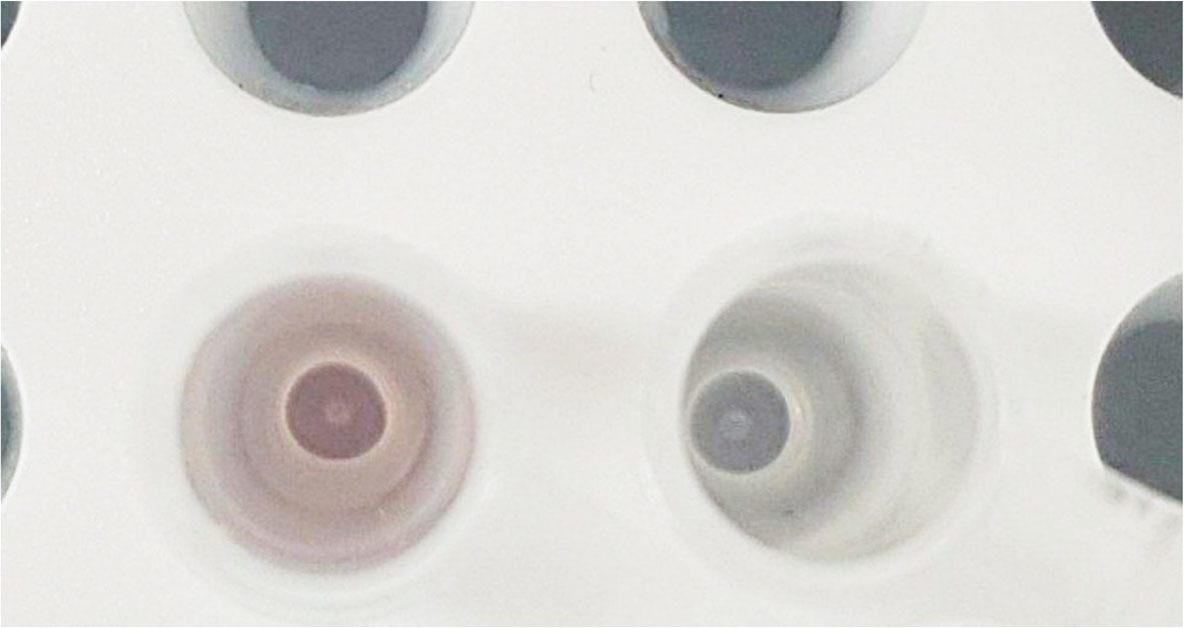
MC-dsRNA interaction results in color change of the MC solution. Color change observed upon addition of 20 μL poly GC dsRNA to a 50 μL of 12.5 μM MC solution in TDW at pH 7 (right). 20 μL of TDW was added as a control for comparison (left)

Considering that the MC form is positively charged, Na + ions in the buffer may hinder the dsRNA-MC interaction. Previous studies also showed that the presence of strong ionic strength can create an ion atmosphere [45] and may weaken interaction with nucleic acids [46]. To test, we used pH 7 TDW as the solvent for MC and examined the spectral change upon addition of synthetic dsRNAs. We found that using TDW results in more prominent hypochromic change at 512 nm (Fig. 2e and f). The absorbance change for the poly GC dsRNA was so dramatic such that the color of the solution was changed from red to pale/colorless (Fig. 3). The large spectral effects accompanied by dramatic color change suggest that spiropyrans can potentially be used as a sensing tool for dsRNAs. Of note, we have also tested SP-dsRNA interaction in TDW, but it still does not show any effect (Additional file 1: Figure S4). Therefore, SP is unlikely to interact with dsRNAs and only the MC form can intercalate between RNA base pairs.

Close examination of the spectral changes revealed lack of the third isosbestic point, which suggests that the main interacting species to dsRNA is MCH+ rather than MC. In addition, the 432 / 512 nm absorbance peak ratio increases linearly with increased dsRNA concentration. This indicates that higher level of dsRNA in the solution results in more MC intercalation and increased protonation to MCH+ to maintain the linear increase in the absorbance ratio (Additional file 1: Figure S5). Since SP does not absorb neither at 432 nor 512 nm, examining changes in the absorbance peak at these two wavelengths is ideal for further analysis of the MC-dsRNA interaction. Overall, our results suggest that the Na + ions may create an ionic “cloud” [47] which prevents MC from undergoing protonation due to the presence of many counter ions in the solution. Consequently, TDW favors protonation, resulting in increased level of MCH+ intercalated with dsRNAs and allowing MC to be more sensitive towards dsRNAs.

We further examined the interaction between MC and dsRNAs using circular dichroism spectroscopy (Additional file 1: Figure S6). We observed an increase in the peak at ~ 260 nm, which indicates increased stacking due to the intercalation of MC in the RNA helix. We also detected a much smaller increase in the ellipticity at ~ 240 nm, which refers to the slight decrease in helicity for both poly AU and poly GC. Both of these results are key indicators of intercalation [48, 49]. Therefore, our circular dichroism analysis further supports that synthetic dsRNAs adopted A-form helix and that MC can intercalate between RNA base pairs to cause increased stacking. We also detected an induced circular dichroism of MC, which provides an additional evidence for the intercalation of MC between RNA base pairs (Additional file 1: Figure S6). Of note, for the circular dichroism measurement, we used TDW as the buffer solution because the interaction between MC and dsRNA becomes stronger in TDW. Our results indicate that poly AU and poly GC both retain their duplex structure and MC can intercalate between RNA base pairs in TDW.

To investigate the length dependency of MC-dsRNA interaction, we prepared poly AU dsRNAs with three different length (10-mer, 20-mer, and 100-mer). When we used equal per base concentration, 20-mer and 100-mer dsRNAs resulted in nearly identical change on the MC spectra (Additional file 1: Figure S7). In other words, 20-mer and 100-mer poly AU dsRNAs have very similar interaction parameters to MC. In contrast, 10-mer poly AU RNAs showed weaker change suggesting that as the RNA molecules become shorter than 20 base pairs, their interaction with MC becomes weaker (Additional file 1: Figure S7). Of note, we used poly AU instead of poly GC for the length dependency test because of the difficulty in synthesizing long poly GC dsRNAs.

### Quantification of MC-dsRNA interaction

**Fig. 4:**
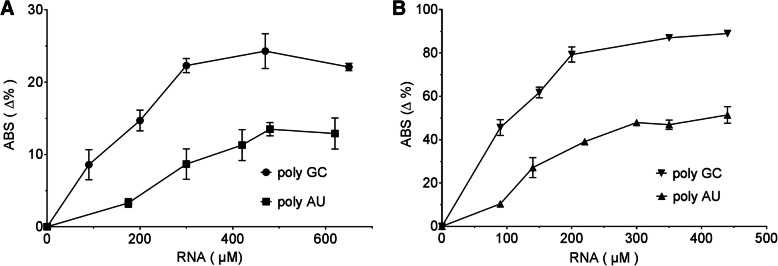
Quantification of the spectral absorbance change to infer dsRNA expression . Quantification of the absorbance change at 512 nm of 12 μM MC Vs. the concentration of poly AU and poly GC in 9 mM Na + ion buffer (**a**) or in TDW (**b**). Average of three replicates are shown and error bars denote s.e.m

For better comparison and quantification of the spectral change induced by dsRNAs, we calculated the percent change in the absorbance peak observed at 512 nm upon addition of different amount of dsRNAs. Together with the normalized 432 / 512 nm absorbance peak ratio, they provide accurate and practical means to sense and even access quantitative information on dsRNA expression. Quantified spectra results are shown in Fig. 4, which shows a clear trend indicating that the change in absorbance of MC increases as the concentration of the RNA is increased and eventually reaches a saturation point. We also found that the absorbance change reaches saturation at much lower RNA concentrations when TDW was used as the solvent. In addition, TDW solvent yielded greater changes in the absorbance compared to that of Na + ion buffer at a given RNA concentration. We determined the binding constants for three different types of dsRNAs in two different buffers used in this study, which are summarized in Table 1.

**Table 1:** MC-dsRNA binding constants. Macroscopic binding constants in 9 mM Na + ions and 1 mM cacodylate pH 7 (buffer 1) and for nuclease free TDW at pH 7 (buffer 2)

ds-RNA	B_max_ (Δ%)	K (M^−1^)	B_max_ (Δ%)	K (M^−1^)
	Buffer 1		Buffer 2	
Poly GC	25.1	7352	90.6	11,111
Poly AU	14.2	3891	52.5	7042
50% GC content dsRNA	~ 20	~ 5000	~ 70	~ 8620

Our calculation confirms the earlier observation that the degree of MC interaction with poly AU is weaker than that with poly GC. For poly GC in low ionic strength condition (TDW), the absorbance change is greater than 90%. In order to dispel any possibilities of alteration in pH driving the observed effect, we confirmed the pH of the systems after dsRNA addition via universal pH indicator, which showed that pH was remained nearly constant at pH 7 (Additional file 1: Figure S8). Our binding constants for both types of synthetic dsRNAs are lower than the ones reported for synthetic and genomic DNA (2 × 10^4^ M^− 1^), calculated using the same spiropyran derivative in the same buffer and pH conditions [22]. One explanation is that dsRNAs form A-form helix, which has narrower and shallow major grooves compared to those of duplex DNA (B-form). Nevertheless, our results clearly indicate that MC, particularly in TDW, show significant and reproducible spectral changes when incubated with dsRNAs.

Characterization of the conversion between MC and MCH+ due to intercalation is summarized in Table 2. The ratio of MC to MCH+ in the intercalated state remains nearly constant with MCH+ to MC molar ratio of 90:10, indicating that MCH+ is the main interacting species. Similar observations were also reported previously for interaction with DNA [22, 50]. There is an increase in the pKa of the system in the presence of dsRNAs, which can be accounted by the increased amount of MCH+ at pH 7. Furthermore, the microscopic binding constant of MCH+ is greater than the observed binding constant for MC because a majority of MCH+ exists in the bound state. These values are calculated from the changes in the absorbance peak at 512 and 432 nm alongside the extinction coefficients for MC and MCH+ as shown in Additional file 1: Figure S3. The corresponding ratios are used to calculate the pKa at pH 7.

**Table 2:** The pKa values and micro/macroscopic binding constants for the MC-dsRNA interaction. The pKa values and micro/macroscopic binding constants calculated in the experiments. From these values, the molar ratio of the binding species for MCH+ to MC was observed to be 90:10. The schematic shows the binding and protonation occurring due to interaction with dsRNAs

Parameter	Value	Schematic
pK_a_^MC^	3.8	
pK_a_^MC-RNA^	7.7	
K_macroscopic_	8.62 × 10^3^	
K_microscopic_	1.13 × 10^4^	
MCH*RNA:MC*RNA at pH 7	~ 90:10	

### Thermal equilibrium analysis of MC-dsRNA interaction

Above, we have used UV to induce isomerization from SP to MC. However, SP can thermally undergo isomerization to become MC in the dark without any UV exposure. Moreover, measuring changes in the thermal equilibrium between SP and MC forms can be used to determine the hydrolysis and the stability of the compound as well as its ability to interact with the ligand [51, 52]. We found that our spiropyran reached thermally induced equilibrium in about 4 h with time constant of 2 h and reached the maximum SP/MC distribution ratio of 74/26. We added 200 μM of dsRNA to each of our samples and monitored the absorbance at 512 and 432 nm. We chose these two wavelengths because absorbance peak at these points showed the greatest changes between SP and MC as well as between MC and MCH+.

**Fig. 5:**
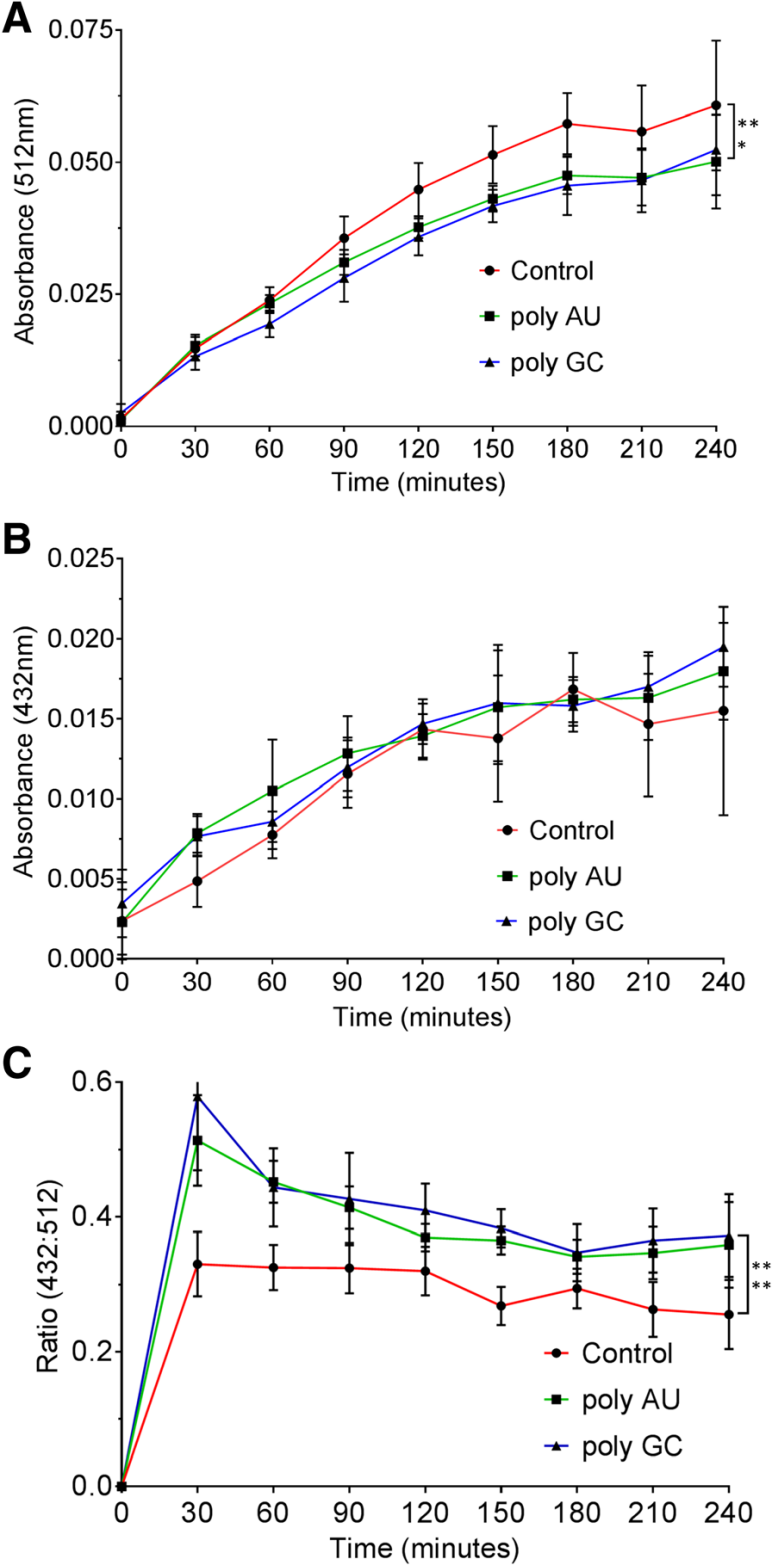
Thermal equilibrium analysis of the MC-dsRNA interaction. **a** and **b** Changes in absorbance at 512 nm (**a**) or 432 nm (**b**) for the three samples (control, poly-AU, and poly-GC) in 9 mM of Na + ion buffer at pH 7. Samples are kept in dark throughout the experiment. **c** The ratio in the absorbance peak at 432 to 512 nm for the three samples. Averages of three replicates are plotted with error bars indicating s.e.m. Statistical tests show that at equilibrium, the absorbance at 512 nm and the corresponding ratios for poly GC (*p* = 0.0138) and poly AU (*p* = 0.0024) were significantly different from that of the control

We found that dsRNA containing samples showed statistically significant decrease in absorbance at 512 nm compared to that of the control sample (Fig. 5a). Addition of poly GC resulted in more prominent change than that of poly AU, which agrees with our previous results. The absorbance at 432 nm was unaffected by the presence of dsRNAs because the drop in MC equaled the gain in MCH+ absorbance spectra (Fig. 5b). Overall, incubation with dsRNAs resulted in increased ratio of 432 to 512 nm absorbance peaks (Fig. 5c). An absorbance ratio of 0.28 is approximately equivalent to the extinction coefficient ratio for MC at the same wavelength without RNAs. This data indicates that there is a shift in equilibrium composition toward more MCH+ when dsRNAs are present, and the observed increase in the 432 to 512 nm absorbance ratio indicates that the protonation of MC to MCH+ by dsRNAs is the main change occurring when MC is mixed with dsRNAs. Therefore, the equilibrium composition of SP to total MC (MC + MCH+) may remain unaffected by dsRNAs and the effect of dsRNA addition is reflected in the decreased 512 nm absorbance peak due to protonation of MC by dsRNAs.

We performed thermal equilibrium analysis only using Na + ion buffer in order to compare our spectral data with our initial characterization and other references. As we expected, all three samples (control, poly AU, and poly GC) stabilized at a roughly constant ratio very quickly. The poly AU and poly GC species adopt a higher thermal equilibrium ratio position and took slightly longer time to reach the equilibrium point than that of the control sample without dsRNAs. Collectively, these results suggest that 432 / 512 nm absorbance ratio is a sensitive measure for detection and quantification of dsRNA amount in a given sample.

### MC can be used to detect dsRNAs isolated from cancer cells

**Fig. 6:**
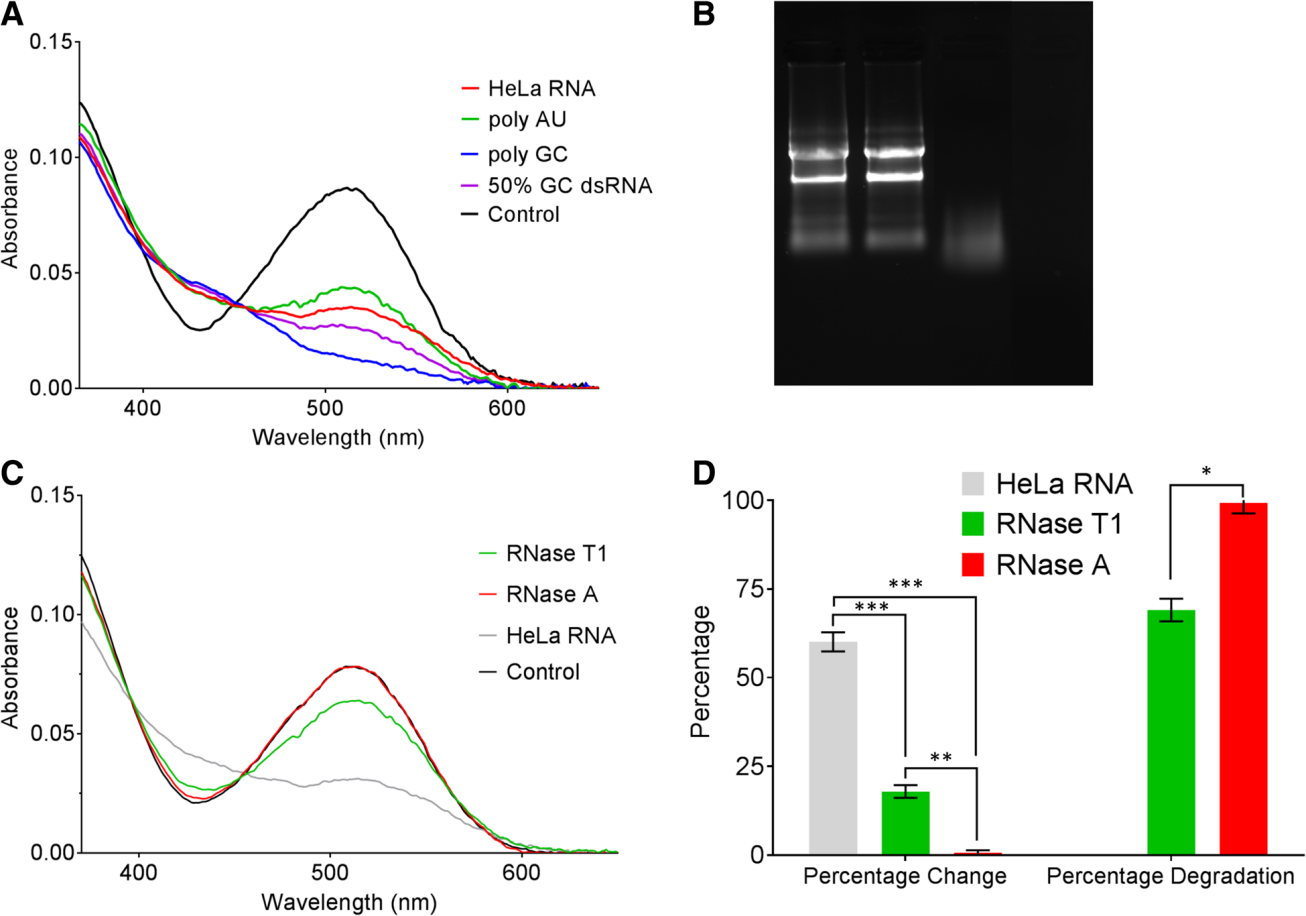
MC can be used to detect cellular dsRNAs isolated from HeLa cells. **a** The absorbance spectra of MC when 350 μM of various RNA samples (synthetic and cellular) were added. **b** Fragmentation of HeLa rRNAs by RNase T1 (lane 3) or RNase A (lane 4) was confirmed using gel electrophoresis. Lane 1 shows the control sample without any RNases and lane 2 shows the control RNAs after incubating it for 30 min in TDW. **c** The effect of RNase treatment on MC absorbance spectra. **d** Quantification of the absorbance spectra upon addition of RNase T1 or RNase A treated HeLa RNAs to MC. Average of three biological replicates is shown with error bars indicating s.e.m. Statistical tests show that both RNase T1 and RNase A treated samples are significantly different from each other and from the control sample

To apply spiropyran to profile cellular dsRNA expressions, we examined whether spiropyran, in particular MC, can detect actual RNAs isolated from cells. We extracted total nucleic acids from HeLa cervical cancer cells and treated them with DNase to remove genomic DNA. Isolated total RNAs were quantified using Nanodrop and 350 μM was added to MC in TDW pH 7. We used 350 μM because this is the concentration where a pure random synthetic dsRNA sample would saturate the MC and result in about 70% change in the absorbance spectra. We observed a significant decrease in the spectral absorbance around 512 nm, which indicates that MC can interact with the base paired regions from the cellular RNAs to become protonated to MCH+ (Fig. 6a). The magnitude of the change for HeLa total RNA is slightly stronger than poly AU, but weaker than 50% GC content 20-mer dsRNA. The major contributor of this effect is likely the ribosomal RNAs (rRNAs), which have extensive secondary structures and constitute over 80% of the total RNA population. Nevertheless, our data indicate that MC can detect genuine RNAs isolated from cancer cells.

To further analyze the MC-cellular RNA interaction, we treated total RNAs from HeLa cells with RNases that have different substrate specificities. First, we used RNase T1, which is an endonuclease that cleaves ssRNA after guanosine residue. RNase T1 treatment would degrade and fragment ssRNAs and hairpin loops but long endogenous dsRNAs would remain intact. Some fragments of rRNAs would also resist RNase T1 as the longest stretch of double-stranded region of rRNA is about 12 bp. However, we expected that most of rRNAs would be eliminated by RNase T1 treatment, essentially enriching dsRNAs in the sample. Therefore, when normalized at 350 μM, the percent absorbance change can be used as a measure to assess the amount of dsRNAs in the sample. Consistent with our idea, we confirmed that RNase T1 digested most of rRNAs and only small fragments remained (Fig. 6b). We also treated total RNAs with RNase A to completely digest all types of RNAs which is also confirmed using gel electrophoresis (Fig. 6b). Of note, as a control, we kept RNAs at 37 °C for 30 min in TDW, which was the duration of the RNase treatment. There, RNAs show minimal degradation indicated by two strong rRNA bands in gel electrophoresis (Fig. 6b). After RNase treatment, we used phenol extraction and washing in order to remove RNases and smaller degraded fragments. We then analyzed the interaction between MC and RNase treated samples using UV-Vis spectroscopy. We found that the spectral absorbance change at 512 nm is decreased for the RNase T1 treated sample (Fig. 6c) compared to the untreated sample. The change observed now can only be accounted by the existence of long dsRNAs left behind in the sample as these RNAs are resistant to RNase T1.

In the RNase A treated sample, the absorbance spectrum became nearly identical to that of the control MC without any RNAs, indicating that nearly all of the RNAs have been degraded and removed (Fig. 6c). Lastly, we examined the possible interaction between individual rNTPs with MC and found that individual RNA nucleotides do not interact with MC (Additional file 1: Figure S9). This result further confirms that the presence of short RNA nucleotides cannot induce protonation of MC. Quantification of the absorbance change is summarized in Fig. 6d, which reinforces our conclusion that RNase A treatment results in no change in the spectra compared to that of the untreated sample while RNase T1 has a moderate effect due to degradation of the single stranded and short hairpin RNAs.

### MC can detect changes in cellular dsRNA expressions in response to DNA demethylation

To examine more clinically relevant context, we tested whether spiropyran can be used to detect changes in dsRNA expression upon chemical treatment. 5-aza-2′-deoxycytidine, commonly known as 5-AZA-CdR or decitabine, is an FDA-approved DNA demethylating agent used to treat MDS patients [53]. Recently, it has been shown that treating HCT116 colorectal cancer cells with a low dose of decitabine results in cell death due to increased level of endogenous dsRNAs and subsequent activation of antiviral signaling [9, 10]. We asked whether spiropyrans could be applied to sense this increase in the overall expression of dsRNAs upon decitabine treatment. Since human cells naturally express some basal level of endogenous dsRNAs as indicated by our analysis of RNAs extracted from HeLa cells, we focused on detecting the increased level of dsRNA expression upon drug treatment. If possible, this information may be used as a predictive marker for drug responsiveness.

**Fig. 7:**
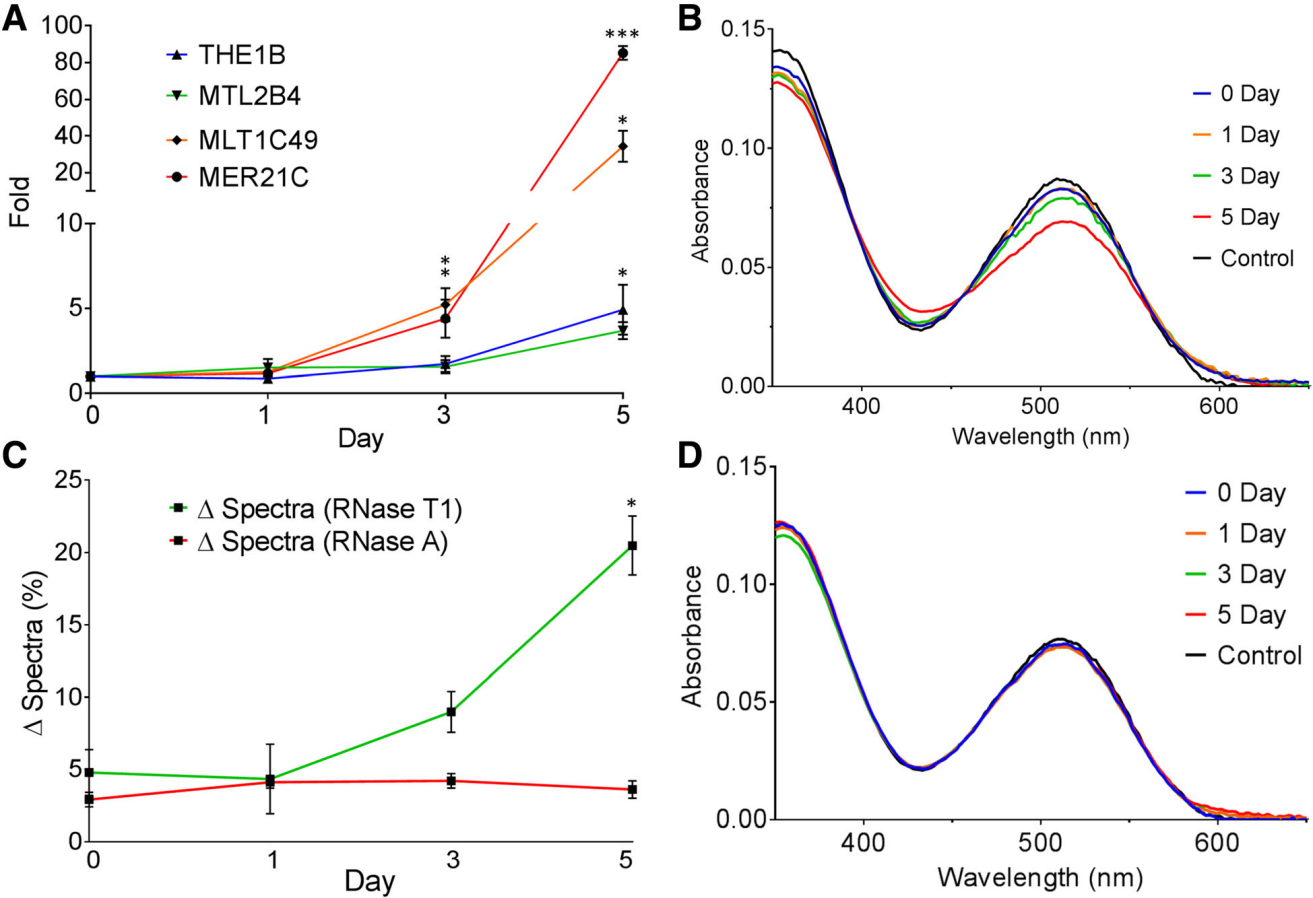
Elevation of dsRNA expression detected using MC. **a** Increased dsRNA expression upon decitabine treatment is confirmed for a number of ERV transcripts using qRT-PCR. The increase was statistically significant for Day 3 and Day 5 samples compared to the preceding days. **b** Changes in the absorbance spectra when RNAs extracted from decitabine treated cells were added to MC. TDW was used as the solvent. **c** Quantified percentage change showing increased dsRNA expression. Day 5 sample is statistically different from day 0 (*p* = 0.0037), day 1 (*p* = 0.0069), and day 3 (*p* = 0.0097). For both (**a**) and (**c**), average of three biological replicates is shown and error bars indicate s.e.m. **d** The absorbance spectra of MC when incubated with HCT116 RNAs treated with RNase A

We treated HCT116 cells with 500 nM concentration of decitabine and collected cells 0, 1, 3, and 5 days after the treatment. We observed a significant decrease in cell proliferation rate 3 days after the treatment, which is consistent with the previous investigation [9]. In addition, using qRT-PCR, we also confirmed increased expression of a selected set of endogenous retroviral element (ERV) transcripts that have been previously shown to be induced by decitabine [9] (Fig. 7a). Of note, these ERV transcripts are noncoding dsRNAs whose expression is induced by decitabine treatment. We then treated these four total RNA samples with RNase T1 followed by phenol extraction to isolate and enrich long dsRNAs. Samples were examined using gel electrophoresis to confirm the RNA integrity and the efficiency of RNase treatment.

We hypothesized that an increased expression of dsRNAs in the sample would result in a greater change in the absorbance spectra of the MC. Upon addition of these isolated dsRNAs to MC, we indeed observed a clear trend of decreasing absorbance around 512 nm region, which is consistent with increase in the overall dsRNA expression (Fig. 7b and c). Day 0 and 1 samples were nearly identical, which is consistent with the qRT-PCR result that ERVs were not yet induced. As ERV expression was induced by day 3, we observed a prominent decrease in the absorbance peak at 512 nm. The degree of the change was greatest for day 5 sample, which is consistent with qRT-PCR result that day 5 sample showed the strongest induction of a number of ERV transcripts such as MER21C (nearly 100 fold compared to the level at day 0). We repeated the experiment three times and obtained highly reproducible and statistically significant changes in the absorbance spectra of MC. Of note, our gel electrophoresis analysis on RNase T1 treated samples showed that rRNAs were not induced by decitabine and all samples have very similar background. As a control, RNase A treated samples were also tested with MC and the results showed no considerable change, indicating complete degradation of all RNAs across all four samples (Additional file 1: Figure S10 and Fig. 7d). A summary of the spectral data is presented in the Additional file 1: Table S1. Collectively, the reproducible change in the absorbance spectra can be accounted by the increase in endogenous dsRNAs.

Our qRT-PCR of individual ERV genes and spectral change of MC show a strong correspondence and correlation with each other. The overall trend in the fold increase in our qRT-PCR results match closely with the percent change in the absorbance spectra of MC. However, considering that the degree of the induction is quite variable among different ERVs and that the human genome encodes approximately 98,000 ERV elements [54], checking the expression change of individual ERV transcripts is not practical and choosing a representative set of ERVs may be misleading. Moreover, it is unclear whether 98,000 ERV elements can all adopt double-stranded secondary structure, which is necessary to activate antiviral signaling. Therefore, our spiropyran offers an alternative diagnostic sensing tool with high reproducibility to predict the cellular response to the DNA demethylating agent used during chemotherapy.

## Discussion

In this study, we have analyzed the interaction between a spiropyran derivative and dsRNAs. The open MC form interacts with dsRNAs with moderate affinity, but the closed SP form does not. We further established that UV-Vis spectroscopy can be used as a simple spectral approach to detect the presence of dsRNAs and analyze MC-dsRNA interactions. Moreover, we applied our spectroscopy-based approach to establish MC as a sensing tool to detect dsRNAs both in synthetic and in actual cellular samples using RNAs extracted from human cancer cells. By employing RNases with different substrate specificities, we have expanded the potential applications of MC to reveal endogenous dsRNA spectral profiles from human cells. Lastly, we applied our approach to report changes in dsRNA expression levels upon treating cells with decitabine, an FDA-approved DNA-demethylating agent used during chemotherapy. Considering that the action mechanism of decitabine induced cell death is through induction of dsRNAs to trigger antiviral signaling, our study suggests that spiropyran can be used to predict the responsiveness to the chemotherapy that relies on similar molecular mechanism.

One limitation of our system is the comparatively low interaction affinity between MC and dsRNAs. Yet, our system allows detection of the total amount of long dsRNAs with minimal sequence specificity and high reproducibility. Owing to the randomness and narrow range for the GC content in the overall sequences observed at the transcriptome-wide scale, we believe that the spectral variations based on AU and GC regions would only have a marginal effect. Therefore, spiropyran can be used to depict the collective expression of long dsRNAs present in cells. Previous attempts to detect RNA expression mostly relied on probes with complementary sequences and thus, their application is limited to individual detection of a few target genes. Moreover, such approach cannot yield information on the total amount of dsRNAs expressed in cells. Considering that immune response proteins recognize double-stranded secondary structure without sequence specificity, our spiropyran-based system provides information that is more relevant. In addition, our spiropyran can be further modified to improve its binding affinity and/or to switch to fluorescent-based detection. For example, it has been shown that amidine-substituted spiropyran showed higher affinity toward DNA [24]. Furthermore, attachment of appropriate fluorescent dye can induce fluorescence resonance energy transfer [55] that can be modulated by MC-dsRNA interaction. Additional modifications are needed in the future to optimize and establish spiropyran as a biosensor for dsRNAs.

In addition to spiropyran, other photochromic molecules have also shown to interact with nucleic acids. When chemically linked, azo-benzenes and diarylethenes have been used for photo-switchable control of DNA [25, 56–61] and RNA hybridization [62, 63]. These molecules all share drastic geometric variations between two isoforms, which make them attractive molecules to control biological systems [64]. The ability of spiropyran to undergo protonation upon intercalation with RNA base pairs is also a key property as a spectral biosensor. Traditional intercalators such as ethidium bromide have been successful in qualitative detection of nucleic acids. Yet, they lack protonated forms which greatly limits their application for quantitative assessments [65]. In addition, spiropyran also provides much greater spectral shifts, which allow us to profile dsRNA expression with a wide range and high reproducibility.

## Conclusions

In conclusion, our work establishes spiropyran as a potential spectral molecular diagnostic tool to detect and assess the overall expression of both synthetic and cellular dsRNAs. We expect that spiropyran based dsRNA detection can be used with a wide range of clinical applications from viral detection, diagnose of human degenerative disease, and prediction of the efficacy of drug treatment from a quantitative and comparative perspective.

## Methods

### Synthesis

Spiropyran used in this study was an Iodo- derivative of the one reported previously [22] with R = CH_2_CH_2_CH_2_(CH_3_)_3_N^+^. The detailed synthesis steps are described in the Additional file 1, and the NMR spectrum of the final compound is shown in Additional file 1: Figure S1. The structure and associated forms referred to are shown in Fig. 1.

### RNA samples

All synthetic duplex RNA samples were ordered as 10 or 20-mers in dialyzed lyophilized powdered form from Bioneer Korea Inc. and re-suspended in a suitable amount of TDW at pH 7 as per the user manuals followed by storage at − 20 °C. 100-mer poly AU RNA was purchased from Santa Cruz Biotechnology and handled in the same way. HeLa and HCT116 RNAs were extracted with TRIsure (Bioline) following the manufacturer’s instruction. The mass of extracted RNA was measured using Nanodrop and the concentration was calculated using specific extinction coefficients at 260 nm (poly AU: ~ 18,000; poly GC: ~ 13,000) for synthetic samples. For cellular RNA samples, an approximate extinction coefficient for double-stranded RNA (~ 15,000) was used for all samples. A previous study has shown that the shifts and error in extinction coefficients for ssRNA and dsRNA mixtures to be in the range of about 3–4% [66]. Based on the percent changes observed when MC was incubated with the enriched dsRNAs, the fraction and expression of dsRNAs in the sample was calculated. Sodium cacodylate buffer was prepared by dissolving sodium cacodylate (Sigma Aldrich) in TDW and adjusting pH to 7.0 by adding diluted HCl. All chemicals were ordered from Sigma Aldrich or Tokyo Chemical Company.

### Characterization

Spiropyran was dissolved in TDW to produce a desired concentration of 12.5 μM and 50 μL of the solution was added to a cuvette. Spiropyran was exposed to UV light (UVL-254 nm, 7 mW lamp) for 5 min to convert it to the open MC form with an absorbance of ~ 0.080 at 512 nm. For the entirety of this experiment, the surrounding was kept dark to prevent any light from converting MC back to SP. 20 μL of RNA solution prepared at a specific concentration was directly added by pipetting to the cuvette containing the spiropyran and spectra were recorded within 2 min. Since the duration of the experiment was much shorter than the time required for the thermal equilibrium of MC itself (4 h), thermal conversion was neglected. The RNA solution was prepared either at 9 mM of Na + ions, 1 mM cacodylate at pH 7 (denoted as Na + ion buffer) or in nuclease free TDW at pH 7 (Ambion). Temperature was kept at 25 °C throughout the duration of the experiment. Spectra were measured with the baseline and background correction in order to scale and establish consistent isosbestic points at ~ 456 and ~ 395 nm. Eppendorf 715 Spectrophotometer with path length of 1 mm was used for UV-Vis spectral analysis with RNase free cuvettes.

Circular Dichroism was done on JASCO-815 using a 1 cm path length in a nuclease free cuvette treated with bleach. Three independent runs were performed with a scanning bandwidth of 1 nm and scan speed of 50 nm / s.

### RNase treatment

For dsRNA sensing studies, RNase T1 (ssRNA specific) and RNase A (ssRNA and dsRNA) were utilized. RNases were dissolved at suitable activity units (see Additional file 1 for details) and incubated with 30 μg of total RNAs for 30 min at 37 °C. Substrate degradation was confirmed with gel electrophoresis on a 1% agarose gel and degraded RNAs and enzymes were then removed through phenol extraction. MC was added to 350 μM of the sample and changes in the UV-Vis absorbance spectra were measured as described above. All samples were normalized to the same mass of RNA (30 μg) before RNase Treatment and then tested at the same concentration post treatment (350 μM) to allow comparison of dsRNA levels in different samples.

### Decitabine treatment and cell samples

500 nM of decitabine (Sigma Aldrich) was treated on 800,000 HCT116 colorectal cancer cells. Total RNAs were extracted using TRIsure and treated with RNase T1 followed by phenol extraction as described above. All the samples were prepared at the same time in identical conditions. 350 μM of RNase T1 treated RNAs were analyzed using MC. For qRT-PCR experiment, cDNA was synthesized using RevertAid reverse transcriptase (Thermo Fisher Scientific) and analyzed using AriaMx qPCR system (Agilent Technologies). Sequences of the primers used in this study are provided in the Additional file 1: Table S2. Detailed method on cell culture and western blotting analysis are available in the Additional file 1.

## Additional file

Additional file 1: Contains supporting information, figures, supplementary tables and additional datareferred to in the main text. (DOCX 2367 kb)

## Abbreviations

dsRNA: Double-stranded RNA
MC: Merocyanine
SP: Spiropyran
Statistical Test Key: * (*p* < 0.05);** (*p* < 0.005);*** (*p* < 0.005)
TDW: Triple distilled nuclease free water
