# Supplementary material for: Spiropyran as a potential molecular diagnostic tool for double-stranded RNA detection
Source: BMC Biomed Eng. 2019 Mar 18;1:6. doi: 10.1186/s42490-019-0008-x (PMC7421392; doi:10.1186/s42490-019-0008-x)
Supplement: Supplementary file 1 — Contains supporting information, figures, supplementary tables and additional datareferred to in the main text. (DOCX 2367 kb) [file 42490_2019_8_MOESM1_ESM.docx]

**Additional file**

**for**

**Spiropyran as a potential molecular diagnostic tool for double-stranded RNA detection** Ahsan Ausaf Ali^1^, Minjeong Kang^1^, Raisa Kharbash^1*^, and Yoosik Kim^1^^*^

*^1^ Dept. of Chemical and Biomolecular Engineering and*

*KI for Health Science and Technology (KIHST),*

*Korea Advanced Institute of Science and Technology (KAIST),*

*Daejeon 34141, South Korea*

*Fax: +82(42)350-3910, E-mail address: raisa@kaist.ac.kr (R.K); ysyoosik@kaist.ac.kr (Y.K)*

**Supporting Experimental Method**

***NMR Characterization and Synthesis***

Experimental procedure for the synthesis of spiropyran compound I4 used in this study.

**Scheme 1.** Synthesis of spiropyran I4.

**1-(3-Iodopropyl)-2,3,3-trimethylindolinium iodide (I1).**

A solution of 2,3,3-trimethylindolenine (1 g, 0.0062 mol) and 1,3-diiodopropane (6.5 g (2.5 mL), 0.0219 mol) in dry acetonitrile (2 mL) was refluxed for 48 hr. After cooling to room temperature, precipitate was filtered off, washed with MeCN and CHCl_3_, and dried. Yield: 1.5 g (56 %), pale grey-yellow solid. ^1^H NMR (400 MHz, DMSO-d_6_) δ 8.01-7.97 (1H, m), 7.88-7.83 (1H, m), 7.68-7.61 (2H, m), 4.52-4.47 (2H, t), 3.46-3.41 (2H, t), 2.87 (3H, s), 2.46-2.36 (2H, m), 1.56 (6H, s).

**1-(3-Iodopropyl)-3,3-dimethyl-2-methyleneindoline (I2).**

Under nitrogen, I1 (0.5 g, 0.001 mol) was suspended in degassed water (120 mL), and finely ground NaOH (1.2 g, 0.03 mol) was added. The reaction was heated at 80 ^o^C for 15 min, then cooled to room temperature. Ethyl ether (130 mL) was added, and the mixture was stirred for additional 1 hr. Aqueous layer was separated and washed with ethyl ether (40 mL) and dichloromethane (40 mL). Organic phases were combined, washed with water, dried over MgSO_4_, and concentrated in vacuum to give I2 (0.30 g, 85 %) as a pink solid. ^1^H NMR (400 MHz, CDCl_3_) δ 7.09-7.04 (2H, t), 6.72-6.67 (1H, t), 6.47-6.44 (1H, d), 3.48-3.44 (2H, m), 2.22-2.15 (2H, m), 1.98-1.90 (2H, m), 1.37-1.33 (2H, d), 1.31 (6H, s).

**1'-(3''-Iodopropyl)-3',3'-dimethyl-6-nitrospiro[(2H)-1-benzopyran-2,2'-indoline] (I3).**

Freshly prepared I2 (0.3 g, 0.0009 mol) was dissolved in dry ethanol (10 mL). 5-Nitrosalicylaldehyde (0.16 g, 0.0009 mol) was added, and the reaction was refluxed under nitrogen for 16 hr. The solvent was removed in vacuum, and the residue was purified by column chromatography on silica gel with hexane/dichloromethane (1:4) as eluent. Compound I3 (0.25 g, 58 %) was obtained as yellow solid after recrystallization from ethanol/chloroform. ^1^H NMR (400 MHz, CDCl_3_) δ 8.02-7.99 (2H, m), 7.21-7.17 (1H, t), 7.11-7.08 (1H, d), 6.96-6.93 (1H, d), 6.91-6.87 (1H, t), 6.75-6.72 (1H, d), 6.65-6.63 (1H, d), 5.89-5.86 (1H, d), 3.35-3.11 (4H, m), 2.28-2.17 (1H, m), 2.11-2.01 (1H, m), 1.28 (3H, s), 1.18 (3H, s).

**1'-(3''-Trimethylammoniopropyl)-3',3'-dimethyl-6-nitrospiro[(2H)-1-benzopyran-2,2'-indoline] iodide (I4).**

The flask containing I3 (0.25 g, 0.0005 mol) was pugged with a rubber septum, and 35 % solution of trimethylamine in ethanol was added to the flask by a syringe. The reaction was stirred at room temperature 48 hr in dark. Ethanol and the excess of trimethylamine were removed by vacuum. The residue was purified by column (dichloromethane/methanol 95/5). The product was suspended in ethyl ether, filtered and dried. Yield: 0.09 g (35 %) ^1^H NMR (400 MHz, DMSO-d_6_) δ 8.27 (1H, s), 8.05-8.01 (1H, d), 7.27-7.24 (1H, d), 7.20-7.14 (2H, m), 6.94-6.91 (1H, d), 6.87-6.83 (1H, t), 6.76-6.73 (1H, d), 6.12-6.08 (1H, d), 3.33-3.30 (2H, m), 3.23-3.18 (2H, m), 3.03 (9H, s), 2.08-1.92 (2H, m), 1.23 (3H, s), 1.15 (3H, s).

See Figure S1 for the NMR spectra.

***Gel electrophoresis***

1% agarose gels prepared using 1x TBE buffer was used to examine the effect of RNase treatment.

***Preparation of RNases***

RNase T1 (Aspergillus oryzae- Worthington Inc) was prepared by dissolving 10 mg in 100 μL of TDW. RNase A (Bovine Pancreas- Worthington Inc) was prepared by dissolving 1 g in 1 mL of TDW. Both RNases were then added in the appropriate volume to allow for the same activity rate. RNases were incubated with 20 μL RNA solutions for 30 min.

Activity units of RNase T1: 346,000 ku/mg

Activity units RNase A: 6,800 ku/mg

Effective activity RNase T1: 3,460,000 U

Effective activity RNase A: 6,800,000 U

***Data analysis***

Data were analyzed by scaling results to the well known isosbestic points (~395 nm and ~456 nm) of MC and MCH+. The key feature of the spectra (512 / 432 nm ratio) is unaffected by this scaling process, which makes this ratio a useful mean to compare different results directly to each other to obtain percentage absorbance changes.

***Cell culture and RNA transfection***

HCT116 cells were cultured in RPMI 1640 (Welgene) supplemented with 10% Fetalgro Bovine Growth Serum (FGR, RMBio). HeLa cells were grown in DMEM (Welgene) supplemented with 10% FGR. Lipofectamine 3000 (Thermo Fisher Scientific) was used to transfect 100-mer poly AU to HCT116 following the manufacturer’s instruction. Cells were harvested 24 hr later and induction of PKR phosphorylation was analyzed through western blotting.

***Western blotting***

Total cell lysates were prepared using the lysis buffer (50 mM Tris-HCl pH 7.4, 150 mM NaCl, 2 mM EDTA, 1% NP-40, 0.1% SDS). 50 μg of protein sample was separated on a 10% SDS-PAGE gel and transferred to a PVDF membrane using the Amersham semidry transfer system. The following primary antibodies were used in this study: PKR (Cell Signaling Technology), phosphor-PKR (Abcam), GADPH (Santa Cruz Biotechnology), and phosphor-eIF2α (Cell Signaling Technology).

**Supporting Figures**


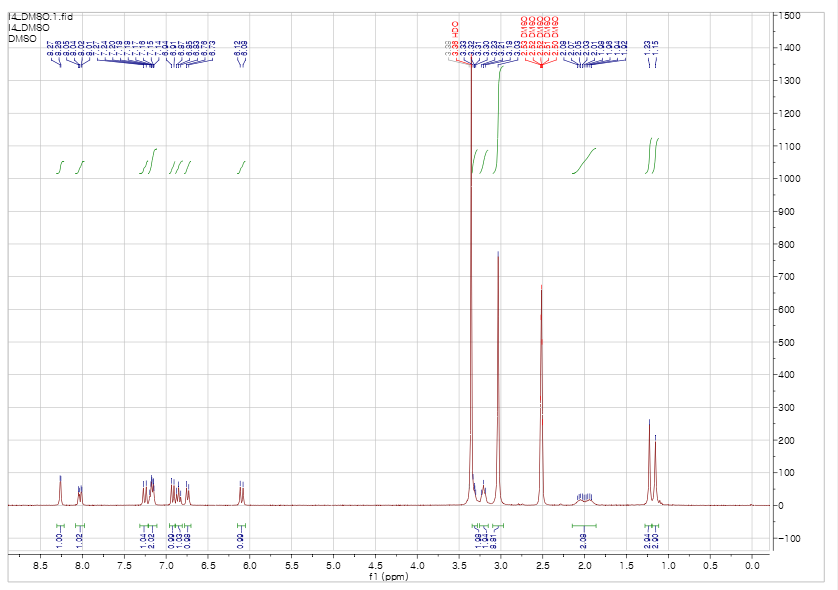


**FIGURE S1. NMR spectrum of the spiropyran.** 1H NMR spectrum of the compound I4 used in this study.


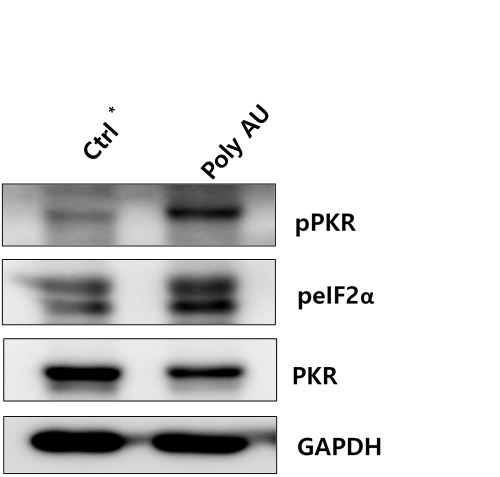


* Ctrl: Lipofectamine treatment only.

**FIGURE S2. Immune response triggered by synthetic dsRNAs**. Transfection of 100-mer poly AU synthetic dsRNAs reconstituted in TDW in HCT116 colorectal cancer cells resulted in increased `phosphorylation of PKR as well as phosphorylation of eIF2α, which can be used as an indicator of active PKR signaling.

**FIGURE S3. Extinction coefficients of all of the species used in our study**. First, we determined extinction coefficient (ε) of SP and MC from the UV-Vis light equilibrium positions and then used the changes observed upon dsRNA addition to calculate ε for rest of the species. The ε for MC (bound), MCH+, and MCH+ (bound) are shown only after a specific wavelength where these values are relevant to our experiment. We can also observe that the principle change occurring upon addition of dsRNA to MC is the red-shift and hypochromic shift accompanied by the protonation of MC to MCH+ in buffer containing 9 mM Na+ ions. Similar, but more dramatic changes were observed when we used TDW as the solvent for MC. The red-shift and hypochromic shift induced by dsRNAs can be used to determine the pKa values for the interconversions between the species.


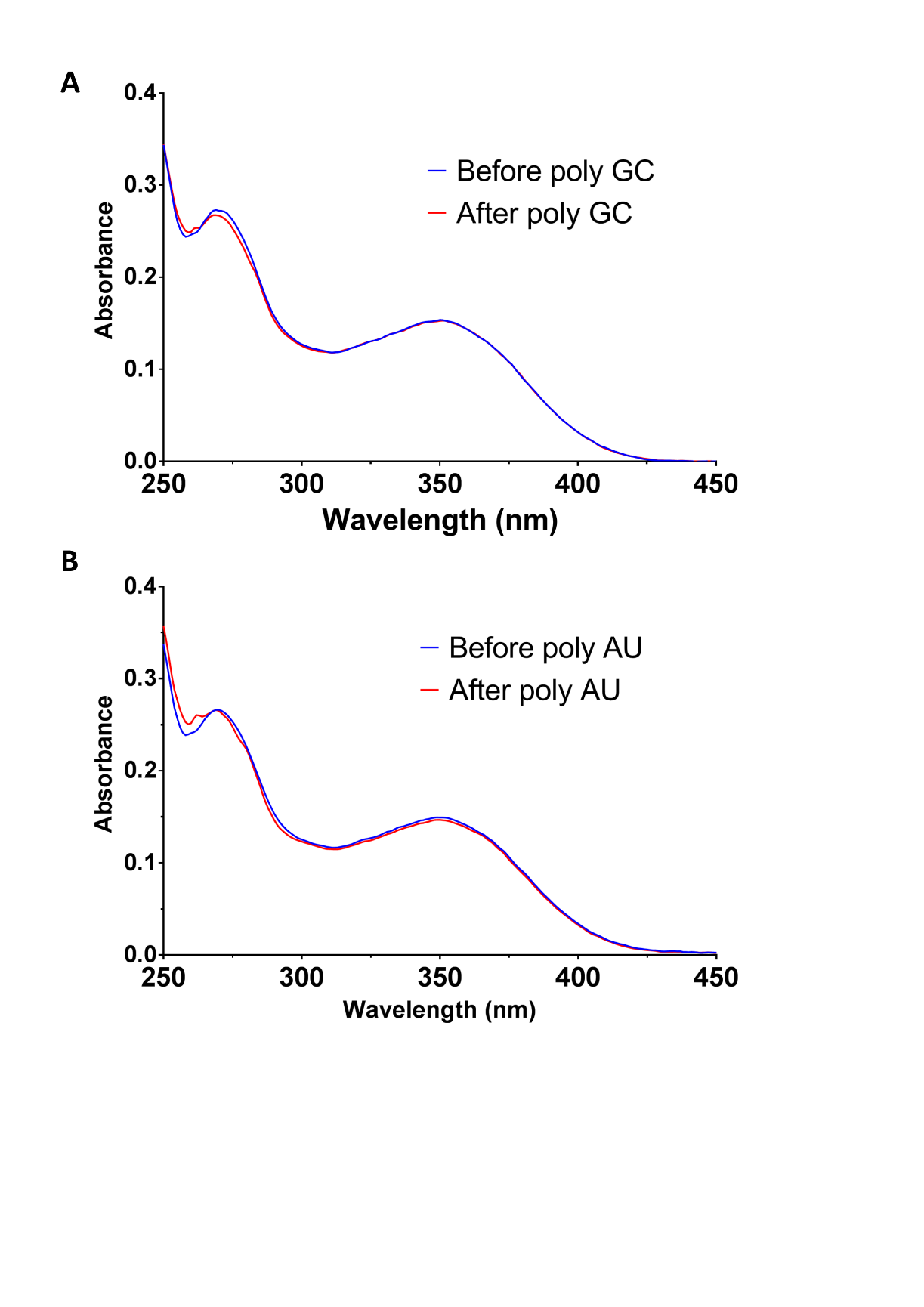


**FIGURE S4. Spiropyran does not interact with dsRNA.** Poly GC (A) and poly AU (B) do not induce absorbance change of SP even in TDW.

**FIGURE S5. The ratio of MC to MCH+ is not affected by dsRNA concentration.** Change in normalized 432 / 512 nm absorbance peak ratio when increasing amount of poly AU or poly GC is added to MC in TDW. The ratio shows a linear trend suggesting that the change in the absorbance spectrum of MC can be used to infer RNA expression levels. This ratio can also be used to examine dsRNAs of any composition, not just poly AU and poly GC. Moreover, this linear trend indicates that the ratio of MC to MCH+ in the RNA bound state remains constant at different concentrations of dsRNAs.


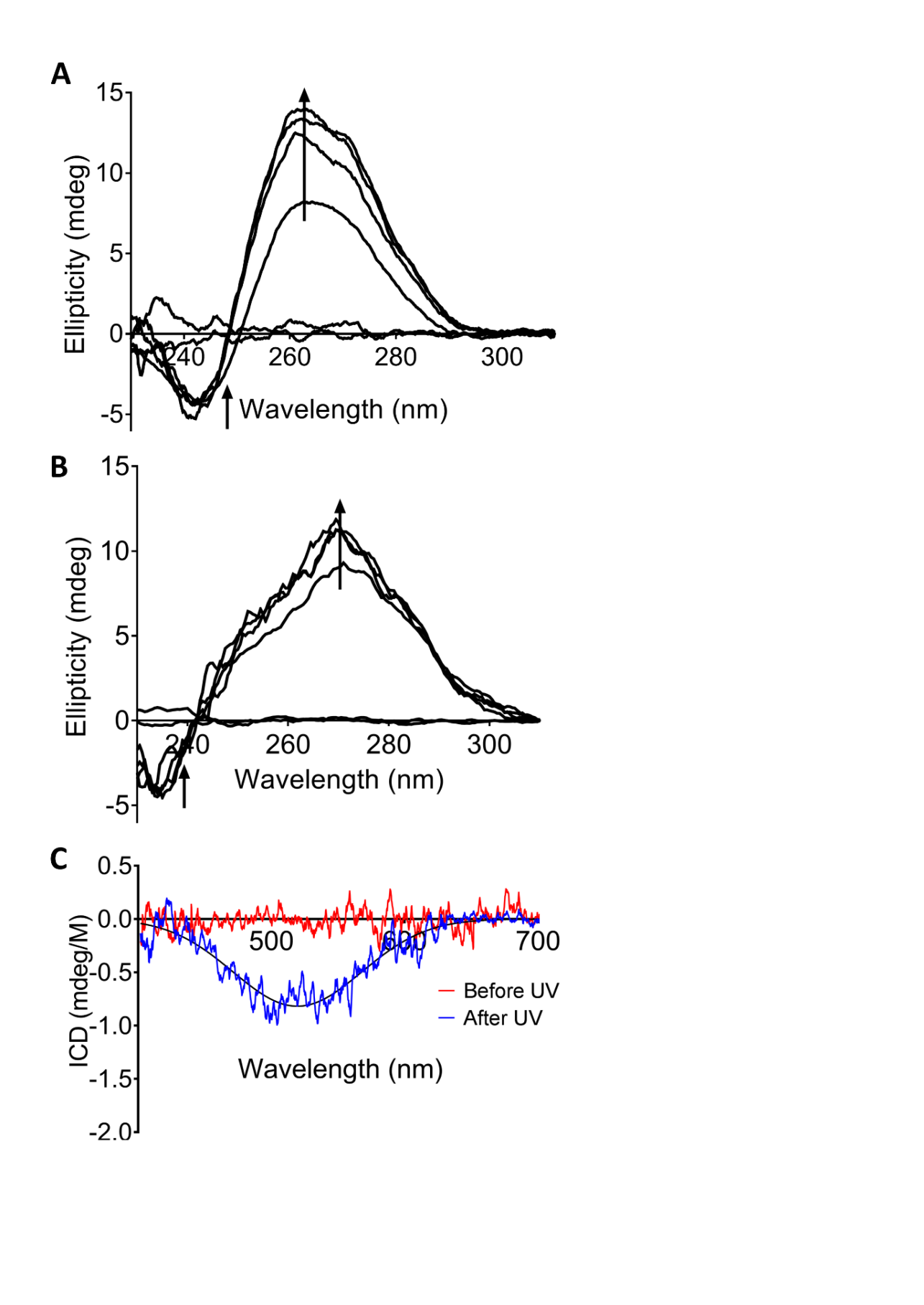


**FIGURE S6. Circular dichroism analysis of MC-dsRNA interaction.** (A, B) Circular dichroism results for poly AU (A) and poly GC (B) dsRNA which confirm the intercalation of MC with dsRNAs. The experiments were conducted in TDW at pH 7. RNA concentration was kept constant at 100 μM and MC concentration was increased from 0 to 100 μM. (C) Induced circular dichroism observed at ~500 nm with a 20-mer dsRNA with 50% GC content. TDW at pH 7 was used as the solvent for both MC and dsRNA.

**FIGURE S7. Length dependency of MC-dsRNA interaction.** Length dependency of MC and dsRNA interaction is examined by using synthetic 10-, 20-, and 100-mer poly AU dsRNAs. Using the same per base pair concentration of 350 μM, 20-mer and 100-mer dsRNAs show nearly identical patterns while 10-mer dsRNA shows slightly weaker effect. This indicates that even at the same per base pair concentration, shorter dsRNAs result in smaller change in the absorbance spectra of MC and that our MC preferably detects dsRNAs 20 base pairs and longer. Thes measurements were obtained using TDW pH7 as the solvent.


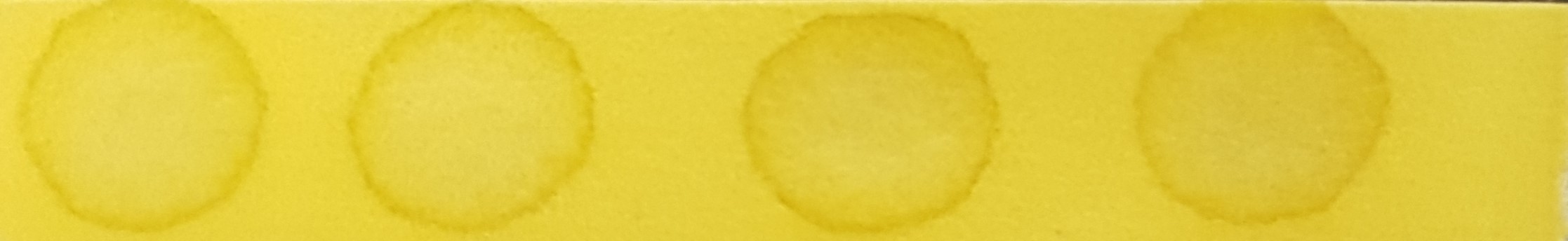


**TDW SP/MC MC-poly AU MC-poly GC**

**FIGURE S8. MC-dsRNA interaction does not change the pH.** The effect of MC-dsRNA interaction on pH of the solution is examined by taking a drop of sample on a universal indicator. No detectable pH variation was observed upon addition of dsRNAs to MC in TDW. The solution retains ~pH 7 in all conditions tested.

**
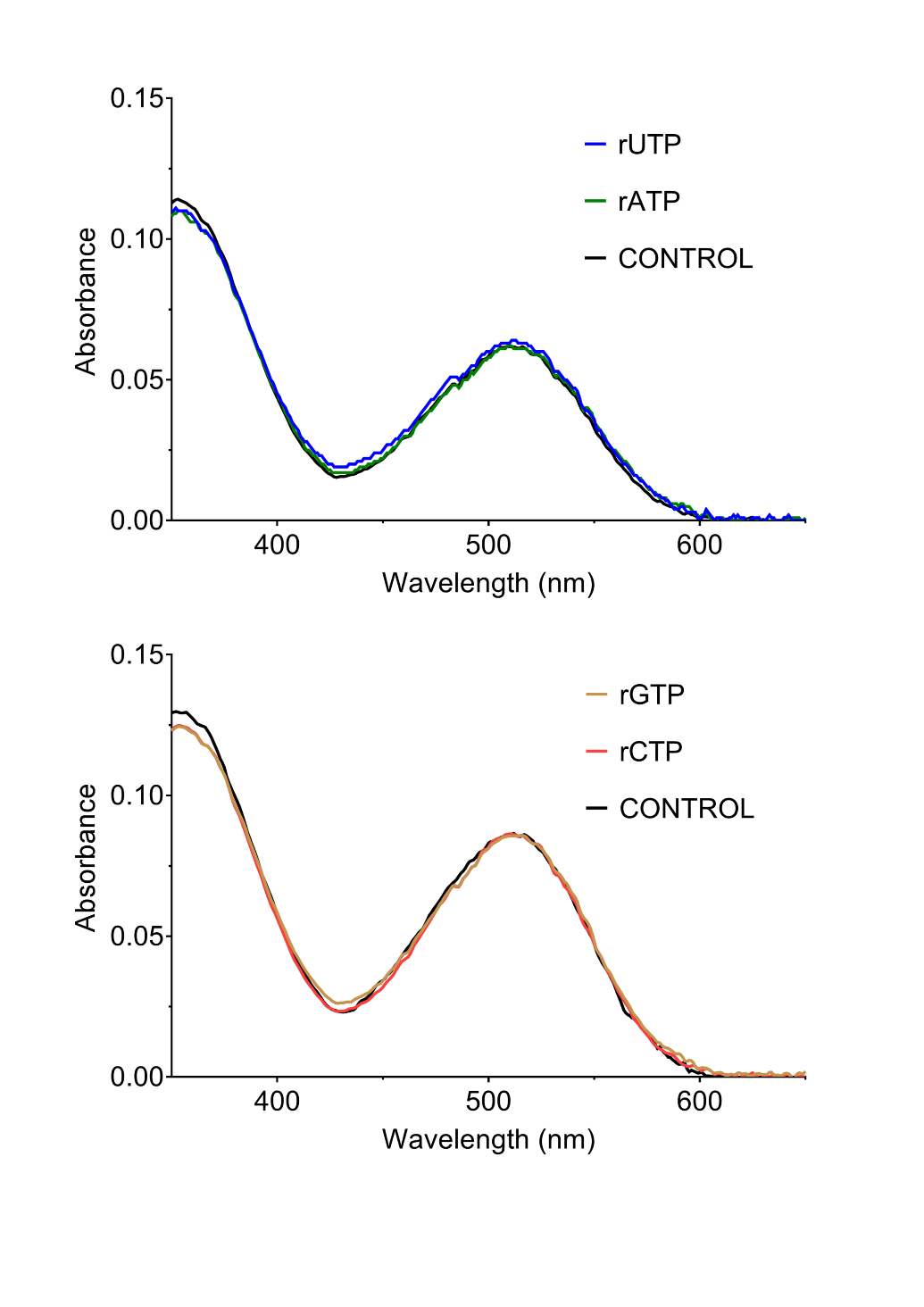
**

**FIGURE S9.** **MC does not interact with individual RNA nucleotides**. Addition of 250 μM of various rNTPs to MC does not affect the absorbance spectrum of the compound, indicating that MC does not interact with individual rNTPs in TDW pH 7.

**FIGURE S10.** **Analysis of RNase treated samples**. Gel Electrophoresis of decitabine treated HCT116 samples (A) followed by their treatment with RNase T1 (B) or RNase A (C).

**Table S1. Analysis of RNase A and T1 treatment on the samples of HCT116 RNA.**

|  | **HCT116 Sample** | | | |
| --- | --- | --- | --- | --- |
| **Measurement** | **Day 0** | **Day 1** | **Day 3** | **Day 5** |
| RNase A (Δ%) | 2.9 ± 0.5 | 4.1 ±0.4 | 4.2 ±0.5 | 3.6 ±0.6 |
| RNase T1 (Δ%) | 4.8 ± 1.6 | 4.3 ± 2.4 | 9.0 ± 1.4 | 20.5 ± 2.0 |
| Change ( T1 – A ) | 1.9 ± 2.1 | 0.2 ± 2.8 | 4.8 ± 1.9 | 16.9 ± 2.6 |
| Change from Day 0  [quantitative] | 0 ± 2.1 ( Baseline ) | -1.7 ± 2.8 | +2.9 ± 1.9 | +15.0 ± 2.6 |
| nc ds-RNA level  [qualitative] | LOWEST | LOWEST | LOW | HIGH |

**Table S2. Primers for qRT-PCR used in the study**

|  | **Primer sequence** | |
| --- | --- | --- |
| **Gene name** | **Forward (5'→3')** | **Reverse (5'→3')** |
| THE1B | TTGATTTTGCAGGCTCACAG | TCCTCCCAAATCTCATGTCC |
| MTL2B4 | CTGCTCCCCACAGTGTCTC | CCAGGTTCAAACTGTTCCAG |
| MER21C | GGAGCTTCCTGATTGGCAGA | ATGTAGGGTGGCAAGCACTG |
| MLT1C49 | TATTGCCGTACTGTGGGCTG | TGGAACAGAGCCCTTCCTTG |
